# Cardiovascular diseases and apical periodontitis: association not always implies causality

**DOI:** 10.4317/medoral.23665

**Published:** 2020-05-10

**Authors:** María Carmen Jiménez-Sánchez, Daniel Cabanillas-Balsera, Victoria Areal-Quecuty, Eugenio Velasco-Ortega, Jenifer Martín-González, Juan J. Segura-Egea

**Affiliations:** 1 DDS, PhD, Doctoral fellow. Department of Stomatology, Section of Endodontics, School of Dentistry, University of Sevilla, Spain; 2 DDS, Doctoral fellow. Department of Stomatology, Section of Endodontics, School of Dentistry, University of Sevilla, Spain; 3 MD, PhD, DDS, Professor. Department of Stomatology, Comprehensive Dentistry Section, University of Sevilla, Spain; 4 DDS, PhD, Associate Professor. Department of Stomatology, Section of Endodontics, School of Dentistry, University of Sevilla, Spain; 5 MD, PhD, DDS, Professor. Department of Stomatology, Section of Endodontics, School of Dentistry, University of Sevilla, Spain

## Abstract

**Background:**

Several studies published in the last two decades have found an association between the prevalence of apical periodontitis (AP) or root canal treatment (RCT) and cardiovascular diseases (CVDs). However, the demonstration of association does not prove by itself the existence of a cause–effect relationship. Two diseases can appear as statistically related without any of them directly affecting the values of the other, resulting in a non-causal relationship. The aim of this narrative review is to summarize the current state of knowledge regarding the association between AP and CVDs, analysing it according to the Hill's causality criteria.

**Material and Methods:**

Epidemiological studies carried out on the association between CVDs and AP or RCT published in English until 8 December 2019 were identified. Forty-four articles were selected and its results were analysed.

**Results:**

Numerous cross-sectional epidemiological studies have found significant relationship between CVDs and AP. The odds ratio values range 1.6 - 5.4. However, other studies have not found significant association. Respect to RCT, some studies found correlation, but others found no association or even found that RCT is a protective factor against CVDs.

**Conclusions:**

The results are inconsistent and a causal relationship between CVDS and endodontic disease cannot be stablished. The risk factors common to both diseases can act as confounding factors, biasing the results. To reach definitive conclusions about the type of association (causal or non-causal) between both diseases, longitudinal epidemiological studies must be carried out to establish the temporal relationship and the dose-response gradient.

** Key words:**Apical periodontitis, atherosclerosis, cardiovascular disease, coronary heart disease, endodontic medicine, root canal treatment.

## Introduction

Cardiovascular diseases (CVD) are a group of disorders related to the heart and blood vessels. CVDs are the number one cause of death globally. Coronary heart disease (CHD) encompasses a range of heart disease whose origin lies in the inability of coronary arteries to supply sufficient blood needed for a given territory of the heart muscle, which results in less oxygen to the heart and greater accumulation of metabolites (1). The clinical manifestations of CHD are basically: 1) Angina Pectoris: the reversible decrease of coronary blood flow produces a myocardial ischemia resulting in acute chest pain, 2) Acute Myocardial Infarction (AMI): a coronary blood flow decrease produces a total myocardial ischemia in an area of heart muscle, with necrosis of myocardial cells and irreversible impairment, and 3) Sudden death. CHD is the major cause of death after 40 years in men and 64 years in women, causing 70% of deaths from 75 years of age. The most common cause of CHD is the blockage of the coronary arteries by atherosclerosis, which is present in more than 90% of cases of CHD. Besides the well-established risk factors for CHD such as smoking, hypertension, high low density lipoprotein (LDL) serum levels, diabetes, gender, obesity, socioeconomic status, and genetic dispositions, chronic inflammatory processes have been considered as potential predictors for arteriosclerosis (2).

Apical periodontitis (AP), an inflammatory process around the apex of a tooth root, is primarily a sequel to microbial infection of the pulp space of teeth. The infectious aetiology of AP and the main role of microbial factors initiation, development and persistence, has been widely documented (3). The prevalence of AP rise 61% of individuals and 14% of the teeth, increasing with patients age (4). Root canal treatment (RCT) the elective treatment for teeth with AP that must be preserved. In Europe, the prevalence of endodontic treatment is estimated around 41% of individuals and 2% - 9% of teeth (4).

AP may not exclusively be a local phenomenon. It is well known that in its non-balanced acute stage spreading of the infection and the inflammatory process to nearby tissue compartments is possible and may bring about severe, but fortunately rare, fatal inflammatory conditions. Moreover, considering the increasing awareness of a potential relationship between persistent, inflammatory disorders of the oral cavity and disease conditions in other organs of the body, acute and chronic manifestations of AP may also be implicated (5).

In the three last decades, several epidemiological studies have found association between CHD and acute myocardial infarction AMI and periodontal disease (6). Apical periodontitis and periodontal disease share similar characteristics: 1) both are chronic infections of the oral cavity, 2) both are polymicrobial infections sharing a common microbiota with predominance of Gram-negative anaerobic bacteria, and 3) elevated cytokine levels may be released systemically from acute and chronic manifestations of both disease processes (7). Similarly, one might assume that AP is associated with the same systemic disorders that are associated with PD (6), such as CVDs.

However, the results of the epidemiological studies should be carefully evaluated. Hill's causality criteria (8) must be those that definitely allow valid conclusions to be drawn about the nature, causal or not, of the association between CVDs and AP.

The aim of this narrative review is to summarize the current state of knowledge regarding the association between AP and CVDs, analysing it according to the Hill's causality criteria.

## Material and Methods

The aim of this study is not to perform a systematic review, however it has been necessary to review the scientific evidence on the relationship between CVDs and AP or RCT. Epidemiological studies carried out on the association between CVDs and endodontic disease published in English until 8 December 2019 were identified and analysed. An electronic search of PubMed, Web of Science and Scopus was conducted using appropriate keywords: (endodontic OR dental pulp OR pulpitis OR apical periodontitis OR periapical granuloma OR root canal treatment OR root filled teeth) AND (cardiovascular disease OR coronary heart disease OR coronary artery disease OR cerebrovascular disease OR atherosclerosis OR atherosclerotic) AND (epidemiological OR cross-sectional OR retrospective OR case-control OR prospective OR longitudinal OR cohort OR clinical trial OR systematic review). Human cross-sectional and prevalence studies, retrospective and case–control studies, prospective and cohort studies, and systematic reviews were included. Animal and experimental studies as well as case reports or case series studies were excluded. Forty-four articles were selected, and its results were analysed.

## Results

- Scientific evidence on the association between cardiovascular diseases and apical periodontitis

At the end of the 20th century, several epidemiological studies were carried out investigating the association between dental infections, including periodontal disease and chronic apical periodontitis, and cardiovascular pathology. Mattila *et al*. [1989] (9) used the Total Dental Index (TDI) to assess the overall state of oral inflammation of patients with acute myocardial infarction (IAM). The TDI analyses the presence of caries, periodontal disease, periapical lesions and pericoronitis, being calculated from the sum of the values recorded for each variable, giving a final score between 0 and 10, with a higher score indicating more severe disease. TDI score was significantly higher in patients with acute myocardial infarction than in controls (OR = 1.3; *p* = 0.004), remaining the association valid after adjustment for age, social class, smoking, serum lipid concentrations, and the presence of diabetes (9). It was concluded that dental infections were a risk factor for coronary events in patients with CHD.

- Prevalence of radiolucent periapical lesions and cardiovascular diseases

Specific scientific evidence about the possible association between CVDs and endodontics has been provided by relatively recent studies (Table 1). It was at the beginning of the 21st century when the first epidemiological studies have been conducted directly investigating the association between cardiovascular diseases and apical periodontitis. Several cross-sectional studies found that radiolucent periapical lesions are significantly more prevalent in patients with CVDs. Patients with AMI exhibited an unfavourable dental state, with significantly higher number of radiological periapical lesions and root-filled teeth, compared with individuals without myocardial infarction (2). Moreover, patients with first attack of angina pectoris or AMI, showed a prevalence of radiographically diagnosed periapical lesions significantly greater than healthy controls (10). Subjects with CHD had higher prevalence of apical periodontitis than healthy controls (OR = 4.37; 95% CI, 1.69-11.28; *p* < 0.05) (11). Likewise, patients with chronic apical periodontitis had a 2.8 times higher risk of developing coronary artery disease (12), and were more likely to have CVDs than subjects without AP by 5.3-fold (13). In a study population from Sweden including 120 randomly selected patients from an epidemiological sample of 1676 individuals, AP was associated with CVD with an OR 3.8 (95% C.I. = 1.18–12.40; *p* = 0.025) (14).

**Table 1:**
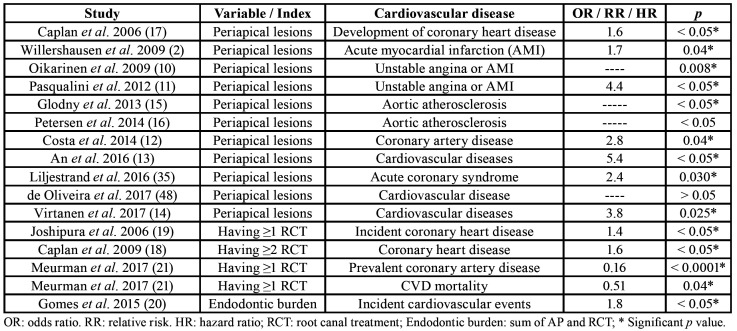
Epidemiological studies analyzing the association between CVDs and apical periodontitis or root canal treatment.

Chronic apical periodontitis has been found to correlate positively with higher aortic atherosclerotic burden (15). The volume of the aortic atherosclerotic burden for patients with at least one chronic periapical lesion was significantly higher than for patients with no periapical lesions (*p* < 0.05) (16).

Few longitudinal studies have investigated the possible association between CVDs and AP. As part of the VA Dental Longitudinal Study, it was investigated whether incident radiographically evident lesions of endodontic origin were related to development of coronary heart disease (CHD). Among patients < 40 years old, incident lesions of endodontic origin were significantly associated with time to CHD diagnosis (*p* < 0.05), after adjustment for covariates of interest, with hazard ratios decreasing as age increased (17).

- Root canal treatment and cardiovascular diseases

The possible relationship between the prevalence of RCT and CVDs has been also investigated (Table 1). Two studies have found association between RCT and CHD, finding that patients with a greater history of RCT were more likely to have CHD compared to those reporting no history of RCT (18,19). The relative risk for incident AMI in patients with root-filled teeth (RFT) was 1.4 times higher (95% C.I. = 1.14-1.67; *p* < 0.05) compared with those without RFT (19). Among patients with 25 or more teeth, those reporting having had endodontic treatments two or more times, had 1.62 (95% C.I. = 1.04 - 2.53; *p* < 0.05) times the odds of prevalent CHD compared with those without RFT (18).

Endodontic burden, calculated as the sum of periapical lesions and RCT sites, has been associated with higher risk of incident cardiovascular events (RR = 1.77; 95% C.I. = 1.04–3.02; *p* < 0.05) (20). However, it has been also shown that having ≥1 RFT is associated with significantly lower odds for prevalent coronary artery disease and lower risk of cardiovascular mortality (21).

The systematic review conducted by Berlin-Bröner *et al*. [2017] (22) concluded that, although most of the published studies found a positive association between apical periodontitis and CVDs, the quality of the existing evidence is moderate-low and a causal relationship cannot be established. Even though a significant relationship between chronic apical periodontitis and atherothrombotic cardiovascular disease has been showed in 11 of 15 analyzed studies, there was a huge methodological heterogeneity.

A recent study by Messing *et al*. [2019] (23) has used epidemiological and genetic approaches, querying the medical and dental re- cords of >2 million patients. A significant association between the presence of endodontic pathology and a history of myocardial infarction, cerebrovascular accident, pacemaker, congestive heart failure, heart block, deep vein thrombosis, cardiac surgery, and, particularly, hypertension, have been found.

- Possible mechanisms involved in the association between cardiovascular diseases and endodontics

**Figure 1:**
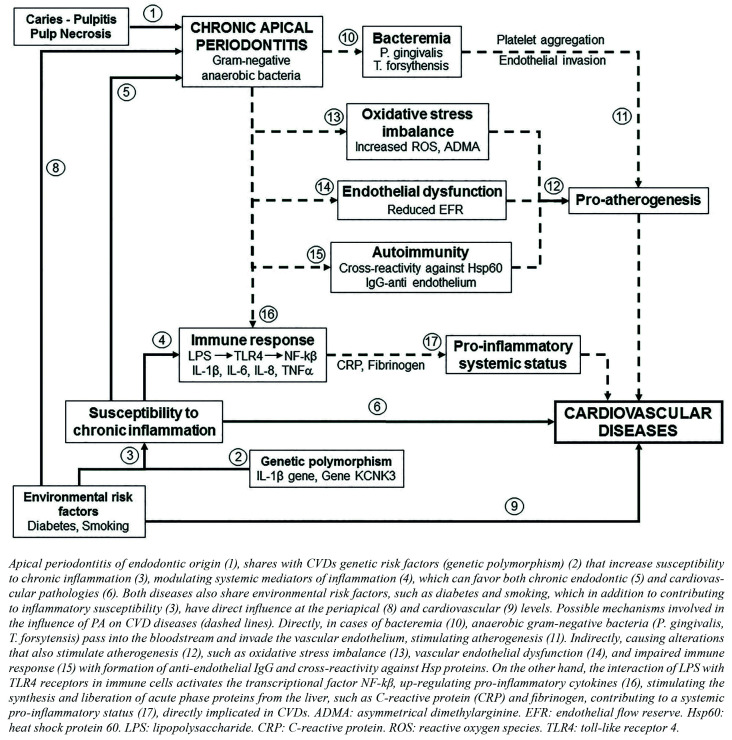
Risk factors common to endodontic pathology and CVDs (continuous lines).

Several pathways linking endodontic infections on CVDs could be suggested (Fig. 1).

Firstly, transient bacteraemia, with spreading of infection from the oral cavity to a distant site, which is colonized by those specific bacteria (i.e. atherosclerotic plaque which is then destabilized). The effect of endodontic bacteria and endotoxins, such as LPS, on inflammatory reactions, haemostatic processes, lipid metabolism, and integrity of the vascular endothelium can explain the association between endodontics and CVDs. From periapical inflammatory lesions, subclinical chronic bacteraemia occur and a regular release of cytokines (protein C-reactive a-antitrypsin, haptoglobin, fibrinogen, thromboxane, IL-1, IL-6 IL-8 and TNF), which pass into the general circulation. Direct implication of endodontic bacteria, passing into the bloodstream during transient bacteraemia on blood vessels, could also explain the relationship between apical periodontitis and cardiovascular diseases. Aortic inflammatory response induced by polymicrobial infection (Porphyromonas *gingivalis*, *Treponema denticola*, *Tannerella forsythia*, and *Fusobacterium nucleatum*) and active invasion of the aortic adventitial layer by *P. *gingivalis**, has been demonstrated in mice (24). The role of *Porphyromonas endodontalis* in atherogenesis is also supported experimentally, since it can directly invade endothelial and smooth muscle cells from human coronary artery and induce matrix metalloproteinase (MMP) expression *in vitro* (25). Atherosclerotic plaques, obtained during carotid endarterectomy in humans, were positive for T. forsytans or *P. *gingivalis** (26). The presence of 23 oral bacterial species, including endodontic bacteria such as *Porphyromonas *gingivalis**, *Porphyromonas endodontalis* and *Prevotella intermedia*, in atherosclerotic plaque samples have been demonstrated (27). Nevertheless, it was unknown if these bacteria had either periodontal or endodontic origin.

Secondly, systemic inflammation arising from the immune-inflammatory periapical response, which could increase the inflammatory burden linked to the onset of atherosclerosis (28,29). The passage of bacterial antigens, such as lipopolysaccharide (LPS) of gram-negative bacteria and lipotheicoic acid (LTA) from gram-positive bacteria, known as pathogens associated molecular patterns (PAMPs), from intra-canal and/or extra radicular biofilms into the bloodstream, could influence the general immune status of the host. PAMPs bind to specific toll-like (TLRs) receptors in immune cells, activating the transcriptional factor NF-kβ, up-regulating pro-inflammatory cytokines, and initiating or sustaining a pro-inflammatory systemic status (30). Pro-inflammatory cytokines (IL-1, IL-6 and TNF-α) stimulate the synthesis and liberation of acute phase proteins from the liver, such as C-reactive protein (CRP), and coagulation factors, such as fibrinogen, that contribute to the injury of tissues remote from periapical lesion. Both fibrinogen and aggregated CRP favour atherothrombotic events. The risk of CVDs would be influenced by increasing the concentration of cytokines, CRP, fibrinogen and sialic acid, which are predictors of CHD, as demonstrated in other cases of chronic infections (31). Radiolucent periapical lesions have been associated with the systemic inflammatory burden and cardiovascular risk determined by high-sensitivity C-reactive protein (hsCRP). In multivariate analysis, periapical lesions were significantly associated with hsCRP levels ≥1 mg/L (OR = 5.1-12.8) independently of the adjustment model. Pariapical lesions also associated with CRP levels >3 mg/L (OR = 4.0), supporting a mechanistic link for cardiovascular diseases in young adults (32). On the other hand, TNF-α and IL-1 increase the binding of low density lipoproteins (LDL) to endothelium and smooth muscle, up-regulating the LDL receptor gene, inducing endothelial dysfunction and smooth muscle cell proliferation (33).

Cross-reactivity or a molecular mimicry between endodontic bacteria and host antigens could be another mechanism. The progression of atherosclerosis could be explained in terms of immune response to stress proteins, i.e. heat shock proteins (Hsp). Hsp are highly conserved stress molecules present in both humans and bacteria. The antibody response to them is implicated in atherosclerosis. The body could not differentiate bacterial Hsp of the Hsp themselves, which contain peptide sequences similar to bacterial, producing an autoimmune response. The levels of antibodies to *Porphyromonas *gingivalis** correlated with IgG antibodies to Hsp60 and Hsp70 in patients with periodontal disease (34). An association between periapical lesions, *Porphyromonas endodontalis*, and serum IgG-class antibodies against it has been demonstrated (35). As in atherosclerosis, endothelial cells express Hsp and there would be cross-reactivity of T cells that can damage the arteries of patients with atherosclerosis (36).

Chronic periapical inflammation has been linked to several alterations at the level of the vascular endothelium. Apical periodontitis is associated to early endothelial dysfunction, documented by the reduced endothelial flow reserve (EFR) (37). Endodontic therapy could ameliorate this early endothelial dysfunction (37). Impaired flow-mediated dilatation (FMD) and greater carotid intima-media thickness have been described in patients with AP. FMD was found to be significantly impaired in patients with AP (mean = 4.9% ± 2.05%) compared with healthy controls (mean = 9.74% ± 2.59%, *p* = .000) (38).

Another possible link between CVDs and apical periodontitis could be oxidative stress, strongly involved in the pathology of atherosclerosis, where a chronic inflammatory process develops in the arterial wall (25). Higher reactive oxygen species (ROS) production have been described in patients with chronic apical periodontitis. Superoxide anions as well as hydrogen peroxide production by PMN from peripheral blood in patients with chronic apical periodontitis, were significantly higher in comparison with controls, whereas superoxide levels significantly decreased after surgical removal of periapical lesions (39). Subjects affected by chronic apical periodontitis presented with higher values of oxidative stress, as determined by oxygen metabolites (d-ROMs) test and biological antioxidant potential (BAP) test, and lower antioxidant potential than control patients (40). Endodontic treatment tended to restore the systemic oxidative balance by 90 days following treatment (40). Asymmetrical dimethylarginine (ADMA) levels, the endogenous inhibitor of nitric oxide synthase (NOS), were significantly overproduced in young women with chronic AP and poor EFR, compared to controls (41).

## Discussion

The review of the scientific literature shows numerous studies which results seem to demonstrate the existence of association between CVDs pathology and endodontics. Nevertheless, an association does not always imply causation. In the last decades, one of the topics that has been given more attention in endodontic research is endodontic medicine. Epidemiological studies have attempted to identify associations between apical periodontitis and endodontic treatment with systemic conditions such as diabetes mellitus and CVDs. On numerous occasions, statistically significant associations between endodontic and systemic diseases have been found. However, to demonstrate a significant association, by itself, is not evidence that there is a cause-effect relationship (42). In many cases, there is a statistical association between two variables without any of them directly affecting the other, establishing a non-causal relationship. On the contrary, there is a causal association between two variables, a risk factor and a disease, when it is shown that the factor really influences the development of the disease, affecting its frequency or intensity (8). The causation criteria defined by Hill attempted to assess whether there is a real causality, providing epidemiologic evidence of the existence of causal relationship or not (42). The existence of significant statistical association, based on the odds ratio, relative risk or hazard risk values, refers to the strength of the association and is only the first criterion.

Numerous cross-sectional epidemiological studies have investigated the relationship between CVDs and AP, but only some of them have determined the strength of the association by calculating the odds ratio values (2,11-13,17,35). The OR values provided range 1.6 – 5.4, giving strength to the association between AP and CVDs.

Respect to RCT, the results are obviously inconsistent. Some studies find correlation between the prevalence of RCT and CVDs (18,19), but other find no association (43), or even other find that RCT is a protective factor against CVDs (21).

The temporal relationship of the association has been scarcely investigated. Although incident lesions of endodontic origin has been significantly associated with time to CHD diagnosis (17), definitively establishing a temporal relationship demands that prospective studies are carried out. Similarly, no study has demonstrated a dose response gradient of the association. No studies have been conducted comparing the prevalence of AP or RCT with the prevalence of CVDs. Only one study has found a significant association amongst the number of RCT and the prevalence of CHD (18).

Another Hill´s criteria are coherence and biological plausibility (8). Biological and medical knowledge, and natural history and biology of CVDs and apical periodontitis, must agree with the proposed association. These criteria do not pose a problem. The association between CVDs and AP does not conflict with the generally known facts of the natural history and biology of both diseases. On the contrary, there are several possible biological mechanisms linking CVDs and AP that makes the existence of an association between the two diseases biologically plausible (Fig. 1). A direct mechanism, bacteremia, by which oral bacteria enter the blood stream and invade the arterial wall, is supported by finding showing the presence endodontic bacteria in atherosclerotic plaque samples (26,27). The indirect mechanisms, such as the pro-inflammatory systemic status induced by inflammatory mediators released from inflamed periapical tissues into the blood stream, oxidative stress imbalance, with high ROS production by PMN and increased ADMA levels, and early endothelial dysfunction with reduced endothelial flow reserve (25), are possible, but there is not enough direct evidence of their involvement. Therefore, more studies must be carried out to reach definitive conclusions.

Finally, another possible explanation of the association found between endodontic and CVDs is that it is spurious, due to the presence of confounding factors that have not been controlled in epidemiological studies (Fig. 1). This would be the case of the existence of risk factors common to both pathologies, such could be smoking habits or genetic polymorphisms (6,44). Genetic polymorphisms, i.e. differences in DNA sequence among individuals, groups or populations, with altered gene expression and functional variations of the encoded molecules, can be another confounding factor very difficult to control. Individuals with specific genotypes could be more susceptible to disease or could present an increase in disease severity. Polymorphisms of genes encoding molecules implicated in immune response are associated with CVDs and, at once, the host immune and reparative responses are essential in the development and healing of periapical lesions. Subsequently, a genetic polymorphism could increase the predisposition to CVDs and, at the same time, increase the susceptibility to apical periodontitis, or delay periapical repair. Polymorphisms in IL-1β gene are associated with both apical periodontitis (44) and the risk of myocardial infarction or stroke (45). The existence of genetic polymorphisms involved, at the same time, in the etiology of CVDs and apical periodontitis, could explain the association between both diseases observed in epidemiological studies.

## Conclusions

To interpret the results of the epidemiological studies about the association between CVDs and endodontic disease they must be taken into account all the causation criteria. OR values, even high and significant, do not indicate by themselves causal associations. The two analyzed diseases, CVDs and AP, share common risk factors, which can act as a confounding factor and pretend the existence of a non-existent cause-effect relationship. To reach definitive conclusions about the type of association between both diseases, causal or non-causal, longitudinal epidemiological studies must be carried out to establish the temporal relationship and the dose-response gradient.

Funding

Daniel Cabanillas-Balsera is research fellow supported by Spanish Ministerio de Educación, Cultura y Deporte (Programa Nacional de Formación de Profesorado Universitario, FPU); Mari Carmen Jiménez-Sánchez and Victoria Areal-Quecuty are research fellows supported by the University of Sevilla (Beca de Personal Investigador en Formación, PIF).

Conflict of interest

The authors declare that they have no conflict of interest. This research received no specific grant from any funding agency in the public, commercial, or not- for- profit sectors.
